# Time trends in socio-economic, urban-rural and regional disparities in prevalence of obesity among non-pregnant women in Lesotho: evidence from Lesotho demographic and health surveys (2004–2014)

**DOI:** 10.1186/s12889-021-10571-9

**Published:** 2021-03-19

**Authors:** Betregiorgis Zegeye, Gebretsadik Shibre, Gashaw Garedew Woldeamanuel

**Affiliations:** 1 HaSET Maternal and Child Health Research Program, Shewarobit Field Office, Shewarobit, Ethiopia; 2 Department of Reproductive, Family and Population Health, School of Public Health, Addis Ababa University, Addis Ababa, Ethiopia; 3 Department of Medicine, College of Health science and Medicine, Wolkite Universitiy, Wolkite, Ethiopia

**Keywords:** Inequality, Obesity, Trends, Women, Lesotho, Global health

## Abstract

**Background:**

The growing rates of obesity in developing countries are alarming. There is a paucity of evidence about disparities of obesity in Lesotho. This study examined socioeconomic and area-based inequalities in obesity among non-pregnant women in Lesotho.

**Methods:**

Data were extracted from the 2004, 2009 and 2014 Lesotho Demographic and Health Surveys (LDHS) and analyzed through the recently updated Health Equity Assessment Toolkit (HEAT) of the World Health Organization. Obesity prevalence was disaggregated by four equity stratifiers, namely education, wealth, residence and sub-national region. For each equity stratifier, simple and complex as well as relative and absolute summary measures were calculated. A 95% confidence interval was used to measure statistical significance of findings.

**Results:**

We noticed substantial wealth-driven (D = -21.10, 95% CI; − 25.94, − 16.26), subnational region (PAR = -11.82, 95%CI; − 16.09, − 7.55) and urban-rural (− 9.82, 95% CI; − 13.65, − 5.99) inequalities in obesity prevalence without the inequalities improved over time in all the studied years. However, we did not identify educational inequality in obesity.

**Conclusions:**

Wealth-driven and geographical inequalities was identified in Lesotho in all the studied time periods while education related inequalities did not appear during the same time period. All population groups in the country need to be reached with interventions to reduce the burden of obesity in the country.

## Introduction

Globally, obesity remains one of the major threats to public health. The emerging burden of chronic non-communicable diseases (NCDs), particularly cardiovascular disease (CVD), diabetes and obesity, threatens the gains in life expectancy made by combating infectious diseases [1, 2]. In the African region, where many of these diseases have long been considered “diseases of affluence”, obesity is becoming increasingly prevalent [3, 4]. Vulnerable populations are experiencing high double-burdens of infectious and chronic diseases. The emerging burden of obesity in sub-Saharan Africa if not appropriately addressed, in the next decades, will create new challenges to health systems and threaten global economic development of African countries [5, 6].

Recent estimates from the World Health Organization suggest that NCDs kill near 45 million people each year, representing 70% of all deaths globally [7]. In Africa, over 115 million people suffer from obesity-related problems and the rates are climbing faster than in just about anywhere else in the world [8]. Available evidence suggests that obesity, together with excessive consumption of fat and salt, are risk factors for occurrence of chronic problems such as cancer, chronic kidney disease, diabetes, stroke and heart disease [9]. Furthermore, it is well-established that obesity has a detrimental effect on reproductive physiology as it reduces fertility and increase the risk of adverse outcomes for mother and child.

Interest for NCDs surveillance had mostly remained the concern of developed countries until the 1990s, when it became evident that the greatest impact of NCDs would be in low- and middle-income countries (LMICs). The 53rd World Health Assembly adopted the “Global strategy for prevention and control of non-communicable diseases” [10]. The WHO has also adopted a strategy to be implemented by nations worldwide [11] to halt the issue. The strategy put an emphasis on stakeholders’ role in working together to address the health impact [12]. As primary prevention, the adoption and implementation of strategies at individual, societal and institutional levels are necessary to effectively prevent obesity and the associated health burdens [13].

Study shows, Lesotho is one of the African countries with higher proportion of women’s overweight (70.8%) and obesity (30.1%) [14]. While several studies in Africa have reported associations of obesity with socioeconomic factors among the general population by assessing both the overall prevalence and the associated potential risk factors [15–19], there is a dearth of studies examining inequalities in obesity prevalence in Lesotho. Specific evidence in terms of population-level disparities in obesity prevalence is important to plan targeted obesity prevention and health promotion intervention and develop policies that can reduce health inequalities while improving health for all [20, 21].

The resolution positioned surveillance as a key objective of a global strategy, by stressing the need for mapping emerging NCDs epidemics and their determinants with particular reference to poor and disadvantaged populations, in order to provide guidance for policy, legislative and financial measures related to the development of an environment supportive of control [10].

This study aimed to address the evidence gap in the extent and over time change of socioeconomic and area-based inequalities in obesity among non-pregnant women in Lesotho between 2004 and 2014.The specific objective of this study was (i) to determine the extent of socioeconomic and area-based inequalities in obesity prevalence among non-pregnant women in Lesotho between 2004 and 2014 and (ii) to examine trends in socioeconomic and area-based inequalities in obesity prevalence among non-pregnant women in Lesotho between 2004 and 2014.

## Methods

### Data sources

The data were derived from three rounds of Lesotho Demographic and Health Surveys (LDHSs) conducted in 2004, 2009 and 2014 and available in the offline version of WHO HEAT software updated in 2019. The offline version of the HEAT is freely available to researchers and policy makers worldwide and was accessed from the WHO web addressed for free at https://www.who.int/data/gho/health-equity/heat-built-in-database-edition.

The HEAT software contains the WHO Health Equity Monitor (HEM) database [22]. The HEM database stores data generated from Demographic and health Survey (DHS) and Multiple Indicator Cluster Survey (MICS) conducted in several low-or-middle income countries including Lesotho. The database allows inequality analysis of about 30 Reproductive, Maternal, Newborn and Child health indicators including obesity among non-pregnant women [22].

The LDHS are nationally representative surveys that collect information on a wide range of topics such as maternal and child health, maternal and child mortality, domestic violence, maternal and child nutrition, and knowledge about transmission of HIV/ADS. They were implemented by the Ministry of Health and Social Welfare (MOHS) with the financial and technical assistance from ICF (Inner City Fund) International provisioned through the USAID (United States Agency for International development) funded MEASURE DHS program. Participants in the LDHS were sampled through a stratified two-stage cluster sampling procedure. In the first stage, Primary Sample Units (PSU) or clusters were selected from sampling frame produced in the recent national population census. Then, in the second stage, households were selected from each selected cluster. .. All reproductive age women (15–49 years) in each selected household were selected for interview, which was 7522, 7786 and 6818 were selected in 2004, 2009 and 2014 surveys respectively. And a total of 7095, 7624 and 6621 reproductive age women were successfully interviewed in 2004, 2009 and 2014 respectively, giving the response rate of 94.3, 97.9 and 97.1% [23–25]. The surveys covered 3143, 3678 and 3154 non-pregnant women age between 15 and 49 in 2004, 2009 and 2014 respectively.

### Selection of variables and measurements

Our outcome variable was prevalence of obesity among non-pregnant women. Non-pregnant women aged 15–49 years with Body Mass Index (BMI) greater than or equal to 30.0 were defined as obese [26–28]. The BMI was calculated as weight in kilograms of women divided by the square of their height in meters [26–28]. Women with BMI of less than 30.0 was coded as not obese (0), whereas, those with BMI of greater than or equal to 30.0 was coded as 1 (obese). We measured prevalence of obesity using four equity stratifiers, namely maternal education status, economic status, place of residence and subnational region. Economic status was approximated through a wealth index. In the LDHS, it was computed based on different household assets and ownerships following a methodology explained here [29] and was coded as poorest, poor, middle, rich and richest. The household assets and characteristics used in the computation of the wealth index were materials household are made of, radio, and television. The wealth index was computed for each of the three LDHS similarly using principal component analysis (PCA) [29]. Educational status was classified as no-education, primary school, and secondary school and above. To increase the number of women in the third category of education, the secondary and higher schooling were merged into one group and doing so helps to accurately estimate inequalities of the obesity by maternal education. Place of residence was grouped as urban and rural. There are ten sub-national regions in the country (Butha-Buthe, Leribe, Berea, Maseru, Mafeteng, Mohales Hoek, Quthing, Qashas Nek, Mokhotlong and Thaba-Tseka).

### Statistical analysis

We assessed the inequality in obesity prevalence using the following steps. First, obesity was disaggregated by the four equity stratifiers: economic status, education, residence and subnational region. Second, the inequality was assessed using four measures of inequality: Difference, Population Attributable Risk (PAR), Population Attributable Fraction (PAF) and Ratio. Difference and Ratio are simple measures, while the other two (PAR, PAF) are complex measures. Ratio and PAF are a relative measures, but the remaining two (Difference, PAR) are absolute summary measures. The choice of summary measures is in compliance with evidence suggesting the scientific importance of adopting both absolute and relative summary measures in a single health inequality study [30]. The main reason being that relative and absolute inequality measures could potentially lead to different, even contrasting conclusions [30], and failing to use both of these methods might lead to biased decisions. Unlike simple measures, complex measures are able to take the entire categories of an equity stratifier into account, and are appropriate measures of inequality when the measured equity stratifiers have more than two categories like maternal education.. On the other hand, simple measures are easy to interpret and understanding, and are appropriate to compare just two categories of a dimension of inequality.

PAR is a complex, weighted measure of inequality that shows the potential for improvement in the national level of a health indicator (obesity prevalence in our case) that could be achieved if all subgroups had the same level of health as a reference subgroup [31]. PAR is calculated as the difference between the prevalence of obesity estimate for the reference subgroup y_ref_ and the national average (μ) of prevalence of obesity: PAR = y_ref_ – μ, where y_ref_ refers to the subgroup with the lowest obesity prevalence estimate for binary dimensions (place of residence) and non-ordered dimensions (subnational region and place of residence). For our study, rural for place of residence and Thaba-tseka (in 2004), and Mokhotlong (in 2009 and 2014) regions for the subnational regions were the references for calculating PAR since these groups had the lowest prevalence of obesity. For ordered inequality dimensions (economic and education status), y_ref_ refers to the most advantaged subgroups. Hence, richest subgroups for economic status and secondary school and above for educational status were the references.

Similarly, PAF is a complex, weighted measure of inequality that shows the potential for improvement in the national level of a health indicator, reduction of obesity in our case, in relative terms, that could be achieved if all subgroups had the same level of health as a reference subgroup [31]. PAF is calculated by dividing the PAR by the national average μ and multiplying the fraction by 100: PAF = [PAR / μ] * 100. Both PAR and PAF takes negative values for adverse health outcome indicators such as obesity, and positive values for favorable indicators. The larger the absolute value of PAR, the higher the level of inequality. PAR is zero if no further improvement can be achieved, i.e. if all subgroups have reached the same level of obesity prevalence as the reference subgroup [31] For education and economic status, Difference (D) was calculated as obesity in the” poorest subgroup” minus obesity in the” richest subgroup”, and obesity in “un-educated subgroup” minus obesity in “secondary education and above subgroup”, respectively. For binary dimension (place of residence), Difference was calculated by subtract the subgroup with the lowest obesity prevalence estimate (rural resident) to the subgroup with the highest obesity prevalence estimate (urban resident). For subnational region, Difference was calculated by subtract region with the highest obesity prevalence estimate to the region with the lowest obesity prevalence estimate. Calculation for Ratio measure was similar with that of the Difference measure except we used subtraction for Difference and division for Ratio. The WHO HEAT version 3.1 software was used for the analysis [31]. The procedures followed for calculating summary measures are discussed elsewhere [30, 31]. The change in obesity prevalence over time was examined by referring to the 95% Confidence Interval (CI) of the different survey years. When the CIs do not overlap, it implies that there is statistically significant difference between the two CIs. If the UIs overlap, then no inequality exists.

### Ethical consideration

The analyses were done using the publicly available data stored in the HEAT software. Ethical procedures were the responsibility of the institutions that implemented and funded the surveys. All DHS surveys are approved by ICF international as well as an Institutional Review Board (IRB) in the country to ensure that the protocols are in compliance with the U.S. Department of Health and Human Services regulations for the protection of human subjects.

## Results

In this study a total of 9975 non-pregnant women were participated. Of them, 6892 (69.1%) were rural residents. Also, 1,393 (13.9%) and 2704 (27.1%) of them were from wealth quintile one (poorest) and quintile five (richest), respectively. Regarding educational status, more than half of the participants (50.4%) had attended secondary school and higher. Whereas, the 4808 (48.2%) attended primary school and 136 (1.4%) were not educated. Overall, 16.5, 17.4 and 20% of women were obese in 2004, 2009 and 2014.

Table 1 shows magnitude and trends of obesity across socio-economic and geographical equity stratifiers in Lesotho from 2004 to 2014. The result shows variation of obesity prevalence between the different categories of the four equity stratifiers. Higher prevalence of obesity was seen among women in the richest (quintile 5) and rich (quintile 4) subgroups compared to that of the poorest (quintile 2) and poor (quintile 1) subgroups, respectively. However, there was either no or small variation in prevalence of obesity across the educational subgroups as the confidence intervals for the subgroups overlap. Urban-rural variation in the prevalence of obesity was seen in 2009 survey, but disappeared in 2004 and 2014 surveys. See Table 1 for the detail.

**Table 1:** Extent and trends of obesity prevalence among non-pregnant women across socio-economic and area-based subpopulation in Lesotho from 2004 to 2014

Dimension of inequalities	2004		2009		2014	
	Estimate (95%CI)	Pop^**n**^	Estimate (95%CI)	Pop^**n**^	Estimate (95%CI)	Pop^**n**^
**Economic status**						
Quintile 1 (poorest)	8.12 (5.53, 11.78)	417	7.14 (4.80, 10.50)	530	6.21 (4.41, 8.68)	446
Quintile 2	9.86 (7.62, 12.68)	562	12.86 (10.24, 16.03)	599	16.05 (12.43, 20.47)	520
Quintile 3	14.81 (11.54, 18.80)	563	12.84 (10.14, 16.13)	654	16.19 (12.96, 20.04)	591
Quintile 4	18.36 (15.00, 22.27)	712	16.23 (13.76, 19.05)	912	25.50 (21.43, 30.06)	761
Quintile 5 (richest)	24.11 (21.24, 27.23)	888	29.93 (26.22, 33.92)	981	27.31 (23.17, 31.90)	835
**Education status**						
No education	13.37 (7.11, 23.73)	60	12.40 (5.63, 25.12)	42	19.12 (6.11, 46.18)	34
Primary school	15.70 (13.91, 17.67)	1838	15.81 (13.88, 17.94)	1735	18.56 (16.04, 21.39)	1235
Secondary school +	17.76 (15.43, 20.35)	1244	19.01 (16.83, 21.39)	1900	20.87 (18.46, 23.51)	1884
**Residence**						
Rural	15.63 (14.19, 17.20)	2390	14.20 (12.80, 15.72)	2471	18.34 (16.30, 20.59)	2031
Urban	19.12 (16.01, 22.66)	753	24.02 (20.65, 27.75)	1207	22.85 (18.79, 27.49)	1123
**Region**						
01 butha-buthe	17.65 (13.82, 22.27)	203	20.38 (16.26, 25.23)	172	24.68 (19.74, 30.39)	189
02 leribe	19.96 (16.22, 24.32)	453	16.65 (13.27, 20.68)	640	20.77 (15.76, 26.85)	497
03 berea	14.27 (11.01, 18.31)	366	22.59 (17.54, 28.59)	511	21.55 (17.82, 25.82)	418
04 maseru	17.09 (14.27, 20.33)	808	18.49 (15.55, 21.84)	964	21.92 (16.87, 27.97)	868
05 mafeteng	19.54 (15.21, 24.73)	358	22.57 (17.50, 28.59)	345	22.69 (17.43, 28.98)	264
06 mohales hoek	17.35 (14.39, 20.77)	315	15.77 (10.20, 23.57)	311	21.02 (15.82, 27.37)	265
07 quthing	16.69 (12.47, 21.98)	198	14.62 (11.12, 18.98)	196	19.70 (13.89, 27.17)	162
08 qashas nek	12.90 (6.97, 22.66)	106	14.67 (10.61, 19.93)	111	16.97 (12.04, 23.39)	93
09 mokhotlong	9.57 (5.45, 16.26)	155	6.99 (4.67, 10.34)	175	8.12 (5.94, 11.01)	167
10 thaba-tseka	8.15 (4.38, 14.65)	178	8.38 (5.18, 13.29)	250	9.47 (6.06, 14.49)	228

### Magnitude and trends of inequalities

Displayed in Table 2 are the extent and over time change of inequality in obesity prevalence among non-pregnant women in Lesotho from 2004 to 2014. While the simple measures (Difference and Ratio) showed wealth and residence related inequality, the complex measures did not. All the inequality measures showed that no education related variation in the distribution of obesity burden. On the other hand, substantial regional inequality remained in all the studied years.

**Table 2:** Magnitudes and trends in socio-economic and area-based inequality in obesity prevalence among non-pregnant women in Lesotho from 2004 to 2014

Dimension	Measure	2004	2009	2014
		Estimate (95% CI)	Estimate (95% CI)	Estimate (95% CI)
wealth index	D	−15.98 (− 20.25, − 11.71)	−22.78 (− 27.53, − 18.04)	−21.10 (− 25.94, − 16.26)
	PAF	0 (− 13.54, 13.54)	0 (− 12.99, 12.99)	0 (− 12.45, 12.45)
	PAR	0 (− 2.23, 2.23)	0 (− 2.26, 2.26)	0 (− 2.48, 2.48)
	R	0.33 (0.20, 0.47)	0.23 (0.14, 0.33)	0.22 (0.14, 0.31)
Education	D	−4.38 (− 12.81, 4.04)	−6.61 (− 16.22, 3.00)	−1.74 (−21.78, 18.28)
	PAF	0 (−9.82, 9.82)	0 (−6.77, 6.77)	0 (−5.68, 5.68)
	PAR	0 (−1.61, 1.61)	0 (−1.18, 1.18)	0 (− 1.13, 1.13)
	R	0.75 (0.28, 1.21)	0.65 (0.15, 1.15)	0.91 (−0.04, 1.87)
Place of residence	D	−3.48 (−7.10, 0.14)	−9.82 (−13.65, −5.99)	−4.50 (− 9.33, 0.33)
	PAF	0 (− 14.60, 14.60)	0 (− 10.60, 10.60)	0 (− 9.59, 9.59)
	PAR	0 (−2.40, 2.40)	0 (− 1.84, 1.84)	0 (−1.91, 1.91)
	R	0.81 (0.65, 0.97)	0.59 (0.48, 0.69)	0.80 (0.62, 0.98)
Region	D	11.81 (5.46, 18.16)	15.60 (9.43, 21.77)	16.55 (10.67, 22.43)
	PAF	−50.52 (−75.46, −25.59)	− 59.86 (−82.31, −37.41)	−59.26 (−80.65, − 37.87)
	PAR	−8.32 (−12.43, −4.21)	− 10.43 (− 14.34, −6.52)	−11.82 (− 16.09, − 7.55)
	R	2.44 (0.89, 4.00)	3.23 (1.72, 4.73)	3.03 (1.89, 4.17)

## Discussion

The study examined the extent and over time change of socioeconomic and regional inequalities in the prevalence of obesity among non-pregnant women in Lesotho using the WHO HEAT. The inequality analysis of the prevalence of obesity showed that findings vary depending on the measures of inequality calculated and equity stratifiers analyzed. We did not see any inequality in the prevalence of obesity by maternal education by all the measures of inequality in all the survey time periods. For wealth and residence stratifiers, inequality existed by the measures of difference and ratio only. Region-driven inequality existed in obesity prevalence in all the three rounds of the Lesotho DHSs.

Lesotho is one of the countries in Sub-Saharan Africa (SSA) with persistently higher obesity prevalence especially among women [32–34]. However, not only is obesity a public health problem in the country, it distributed unevenly across different population subgroups. In this study, obesity burden was more pronounced among the richest wealth quintile than that of the poorest wealth quintile in each of the three Lesotho DHS time points as shown by the Difference measure. However, we did not identify wealth-driven inequality through the complex measures, namely PAR and PAF. The contradiction in the conclusion arrived by the two methods, i.e., simple and complex measures, amounts to the fact that simple measures (difference in this case) compared only the two extreme wealth categories (poorest vs. richest) while the PAR and PAF take into account the entire wealth distribution (all the five categories of wealth) to show the wealth related inequality [30, 31]. As the disaggregation result supports (Table 1), difference revealed that obesity was more concentrated among women in quintile 5 than in quintile 1.

Previous studies in Malawi [35] and Bangladesh [36–38] documented comparable findings which are higher burden of obesity among non-pregnant women in her economic class. This might be due to sedentary lifestyle or occupation that not need intensive labor usually common among individual in higher socioeconomic class, and consume over due to higher purchasing capacities [35, 39–42]. However, another evidence by Rolls BJ (2009) reported that higher obesity in lower socioeconomic groups that might partly due to women in low socioeconomic status most commonly seen when to purchase and consume low quality and unbalanced food [43]. Whoever becomes obese, there is higher risk for chronic disease such as type 2 diabetic mellitus, ischemic heart disease and stroke [44]. Moreover, obesity related premature death and loss of healthy years are seen among low socioeconomic groups [45].

Since simple measures such as difference cannot show the full picture of obesity burden across the entire rugs of wealth index, we calculated PAR and PAF, and reached a conclusion that wealth inequality of obesity did not exist in all the three Lesotho DHSs. This means that the national prevalence of obesity and obesity prevalence among the poorest subgroup (the subgroup with the lowest obesity) are roughly the same. This further means that the average obesity in the country could not be decreased by lowering obesity among the four wealth quintiles (quintiles 2 to 5) to a level in the poorest quintile (quintile 1). The lack of similar evidence on this wealth-based inequality measured through PAR and PAF makes it difficult to compare and contrast our findings.

Similar to the pattern of wealth related inequality discussed above, we observed urban-rural gap in obesity by the simple measures of difference (in 2009 only) as well as ratio. The inequality occurred to the favor of women in rural settings. Similar finding were reported in previous studies in Malawi [35], Algeria and Tunisia [46], Botswana [47], African countries [48] and low and middle income countries [49]. Urban-rural difference in obesity prevalence with higher burden of obesity among urban residents usually attributed by sedentary lifestyle such as consumption of high caloric fast food, and sugar-sweetened beverages as well as decreasing physical activities as documented by previous studies in several African countries [35, 48, 50]. For instance, the laborious nature of occupation in rural residents as compared to their counter urban residents lead them to less likely to accumulate fats in their body [51–53]. Another justification for higher obesity prevalence among urban residents might be due to non-availability of natural foods or unavailability of non-traditional foods and increased the attraction of western products (may be seen as a status symbol) the urban communities are high likely to consume such sedentary foods and then to be obese than rural [54]. This sometimes worsened by cultural perception in Africa that large body is a sign of wealth and estimable [55].

However, complex measures (PAR and PAF) did not reveal obesity burden variations between urban and rural settings with respect to the national average. The explanation for this contradictory result between the simple and complex measures is that, PAR and PAF measured the inequality by comparing the obesity burden in the rural setting (the one with lower obesity burden compared to urban) to the overall national level burden of obesity. The null (zero) value for these measures therefore indicated that obesity in the rural area was similar to that of the country. See the method section for interpretations of PAR and PAF as well as the other measures.

The study also demonstrated marked inequalities in obesity prevalence that disfavored those living in regions such as Leribe (in 2004), Berea (in 2009) and Butha-buthe (in 2014). The obesity prevalence in Lesotho in 2004 could be reduced by between 26 to 76% if obesity burden in the regions had been reduced to a level in Thaba-tseka region. Similarly, the national average of obesity would have been reduced by 60 and 59% respectively in 2009 and 2014 had the respective obesity burden was reduced to levels in regions with the smallest obesity prevalence) (Table 1). This glaring regional variation deserves appropriate health education campaign to create awareness about the bad health consequences of obesity. Our finding is in concordance with prior evidence in Lesotho that showed obesity prevalence varied based on geographic region and ecological zones [25]. Other studies also reported geographic based variations in obesity prevalence [56–58]. Differences between regions in the distribution of resources which helps to promote active living and consumption of healthy foods might contribute to the differential occurrences of obesity across the various regions of a nation [59]. Moreover, variations in socioeconomic conditions between regions could underlie subnational region inequalities in obesity prevalence [57]. For instance, a study in Spain [58] showed that variation in education status between subnational regions was responsible for regional differences in BMI within the country. Similarly, findings in Turkey [60], Canada [61] and Europe [62] revealed that unequal distributions of socioeconomic position of people among regions are responsible for observed inequalities in several health problems.

The inequalities in wealth, residence and region of obesity had not shown sign of improvement over time. Evidence on the overtime dynamics of variations in obesity allows researchers and policy makers to take lesson on how to formulate future interventions to eliminate the variations. In Lesotho, the variations of obesity across wealth and geography remained unchanged over the period of a decade. This could partly reflect that much policy attention was not dedicated to the problem and its uneven distribution in the population.

Interestingly, we did not appreciate significant variation of obesity by the educational status of the mother. That is, by both the simple and complex measures, obesity level did not differ by whether or not a woman is educated. Our finding that obesity did not vary by maternal education is at odds with available literatures [63, 64]. However, our finding contradict with previous studies that reported increased the odds of obesity with increased educational attainment [65–67]. The possible reasons could be that educated women usually are wealthy [68] and commonly living in urban areas where risk factors for obesity are prevalent [69]. Other studies reported that education has positive influence to reduce obesity due to the fact that education create greater opportunity to access health related information, to clearer perception of risks associated with life style, and to have better self-control in life management [70]. The possible explanation for the lack of educational variation of obesity could partly explained by women in all education groups might be reached similarly with obesity minimizing interventions [63].

The study has raised some relevant research and policy implications. First, the adoption of simple and complex inequality measures is important to investigate inequalities from different perspectives. It is likely that simple and complex measures could result in different and even contradicting findings [71]. Researchers who are interested in equity analysis of a health care indicator are required to adopt the methods in a study. Second, interventions aimed at preventing obesity need to reach all the subpopulations irrespective of their place of residence, economic status and regions. The SDG’s “leaving no one behind” entails that all population groups in a country should receive proper amount of attention with respect to both favorable and unfavorable health indicator [72]. Moreover, all subgroups of population in a particular nation have equal rights to access to interventions that aim to reduce obesity as undesired health problems [72].

The study has a few strengths. First, we assessed the inequality in obesity based on the HEM database of the WHO. The database is handled and produced by experts in the area, and this could have improved the quality of the paper. Also, we used the recently updated version of the HEAT software (2019 update), and we were able to reflect relatively current obesity picture in the country. However, the study has some imitations. The study used LDHS and findings from the surveys could not be generalized to geographical areas other than regions as well as urban and rural settings. In addition, the study did not decompose and identify the reasons that underlie the observed obesity inequality in the country. Future inequality studies need to explore the factors underpinning the unequal distribution of obesity across various dimensions of inequality using a decomposition technique.

## Conclusions

The study revealed, by the simple measures of inequality, wealth-driven, urban-rural and subnational regional inequalities in obesity prevalence among non-pregnant women. The inequality occurred to the favors of poor women, residing in rural settings and some regions such as Thaba-tseka. The inequalities remained relatively constant throughout the study period. Interestingly, however, no education related inequality was identified in all the LDHSs. Governments of the country and other concerned stakeholders need to work on ways to reach all population with appropriate interventions to alleviate the burden of obesity.

## Data Availability

The datasets generated and/or analyzed during the current study are available in the WHO’s HEAT version 3.1 [https://www.who.int/gho/health_equity/assessment_toolkit/en/].

## Abbreviations

BMI: Body Mass Index
D: Difference
DALY: Disability Adjusted Life Years
HEAT: Health Equity Assessment Toolkit
LDHS: Lesotho Demographic and Health Survey
PAF: Population Attributable Fraction
PAR: Population Attributable Risk
PSU: Primary Sampling Unit
R: Ratio
SDGs: Sustainable Development Goals
WHO: World Health Organization
